# Clinical Utility and Safety of a Novel Ultraslim Catheter‐Type Peroral Cholangioscope/Pancreatoscope: A Single‐Center Case Series

**DOI:** 10.1002/deo2.70388

**Published:** 2026-07-28

**Authors:** Hiroyuki Kojima, Shuntaro Mukai, Atsushi Sofuni, Takayoshi Tsuchiya, Reina Tanaka, Ryosuke Tonozuka, Kazumasa Nagai, Yukitoshi Matsunami, Hirohito Minami, Noriyuki Hirakawa, Kyoko Asano, Kento Shionoya, Kazuki Hama, Takao Itoi

**Affiliations:** ^1^ Department of Gastroenterology and Hepatology Tokyo Medical University Tokyo Japan; ^2^ Department of Clinical Oncology Tokyo Medical University Tokyo Japan

**Keywords:** pancreatobiliary disease, peroral cholangioscopy, peroral pancreatoscopy, ultraslim catheter‐type peroral cholangioscope, ultraslim catheter‐type peroral pancreatoscope

## Abstract

Peroral cholangioscopy (POCS) and peroral pancreatoscopy (POPS) enable direct intraductal visualization for pancreatobiliary disease diagnosis and management. Nonetheless, conventional devices have relatively large diameters, limiting their use in narrow ducts. This single‐center, retrospective, observational study aimed to evaluate the feasibility and safety of a novel ultraslim catheter‐type POCS/POPS (UCPOCS/UCPOPS) (distal outer diameter: 2.3 mm) in 12 patients who underwent UCPOCS or UCPOPS from November 2022 to August 2024. UCPOCS was performed in eight patients with bile duct stones (*n* = 3), biliary strictures (*n* = 2), intrahepatic cholangiocarcinoma (*n* = 1), post‐hepatectomy biliary bleeding (*n* = 1), and acute cholecystitis requiring endoscopic naso‐gallbladder drainage (*n* = 1). UCPOPS was performed in four patients for postoperative pancreatic duct evaluation (*n* = 2) and intraductal papillary mucinous neoplasm assessment (*n* = 2). Technical success, defined as successful scope insertion into the target duct, was achieved in all cases. Clinical success was achieved in 11/12 cases (91.7%), including diagnostic clinical success in 10/11 diagnostic cases (90.9%) and procedural utility success in 1/1 procedural utility case (100%). The smallest successfully visualized duct measured 1.8 mm in diameter based on fluoroscopic estimation at the intended insertion site. One patient developed mild post‐endoscopic retrograde cholangiopancreatography pancreatitis, which resolved with conservative management; no other adverse events were observed. The median total procedure time and scope insertion time were 42 and 7 min, respectively. These findings indicate that the UCPOCS or UCPOPS is a feasible and safe intraductal visualization tool for pancreatobiliary diseases, particularly in patients with narrow ducts or challenging anatomy.

**Trial Registration**: N/A.

## 1 Introduction

Peroral cholangioscopy (POCS) and peroral pancreatoscopy (POPS) are widely used to evaluate indeterminate biliary strictures, difficult bile duct stones, and pancreatic duct lesions. These techniques enable real‐time intraductal visualization, complementing fluoroscopic imaging and improving diagnostic and therapeutic management of pancreatobiliary disorders [1, 2]. The value of direct intraductal visualization has been established through early and subsequent studies, and POCS‐guided assessment of biliary strictures and cholangioscopy‐assisted lithotripsy are now integral to contemporary endoscopic practice [1, 3].

Recent advances in cholangioscopy systems, including digital single‐operator platforms, have expanded the clinical applications of POCS. Accordingly, guidelines from the Japanese Society of Hepato‐Biliary‐Pancreatic Surgery highlight the role of POCS in evaluating indeterminate biliary strictures and managing difficult bile duct stones when conventional endoscopic retrograde cholangiopancreatography (ERCP) is insufficient [4]. Evidence‐based guidelines from the Japanese Society of Gastroenterology also support endoscopic approaches, including cholangioscopic evaluation, in the management of biliary diseases [5].

POPS has also been increasingly used to evaluate pancreatic ductal lesions, such as intraductal papillary mucinous neoplasm (IPMN), for which direct intraductal visualization provides clinically meaningful information beyond cross‐sectional imaging or pancreatography alone [1, 6].

Despite these advances and guideline endorsements, conventional POCS and POPS scopes typically have outer diameters of approximately 4 mm, limiting stable insertion and visualization in narrow ducts or surgically altered anatomy. To address this limitation, the novel ultraslim catheter‐type peroral cholangioscope (UCPOCS)/pancreatoscope (UCPOPS) (DRES Slim Scope; Japan Lifeline, Tokyo, Japan), with a maximum outer diameter of 2.3 mm, was developed. UCPOCS/UCPOPS enables direct intraductal visualization in ducts difficult or impossible to access using conventional scopes.

The present study aimed to evaluate the clinical feasibility, safety, and procedural outcomes of UCPOCS/UCPOPS in biliary and pancreatic diseases, focusing on performance in narrow ducts and complex anatomical situations.

## 2 Procedure or Technique

This single‐center retrospective observational study was conducted on patients who underwent POCS (UCPOCS) or pancreatoscopy (UCPOPS) using an ultraslim catheter‐type scope for diagnostic or procedural assistance in pancreatobiliary diseases at Tokyo Medical University from November 2022 to August 2024. Patients were included if they were aged 20 years or older and underwent UCPOCS or UCPOPS at our institution during the study period for diagnostic or procedural purposes related to pancreatobiliary diseases, based on clinical judgment. Patients who declined participation after public notification of the study and provision of an opt‐out opportunity were excluded. The study protocol was reviewed and approved by the Institutional Review Board of Tokyo Medical University (approval number: T2024‐0098). The requirement for the acquisition of informed consent from patients was waived owing to the retrospective nature of this study.

### 2.1 Device Description

**FIGURE 1:**
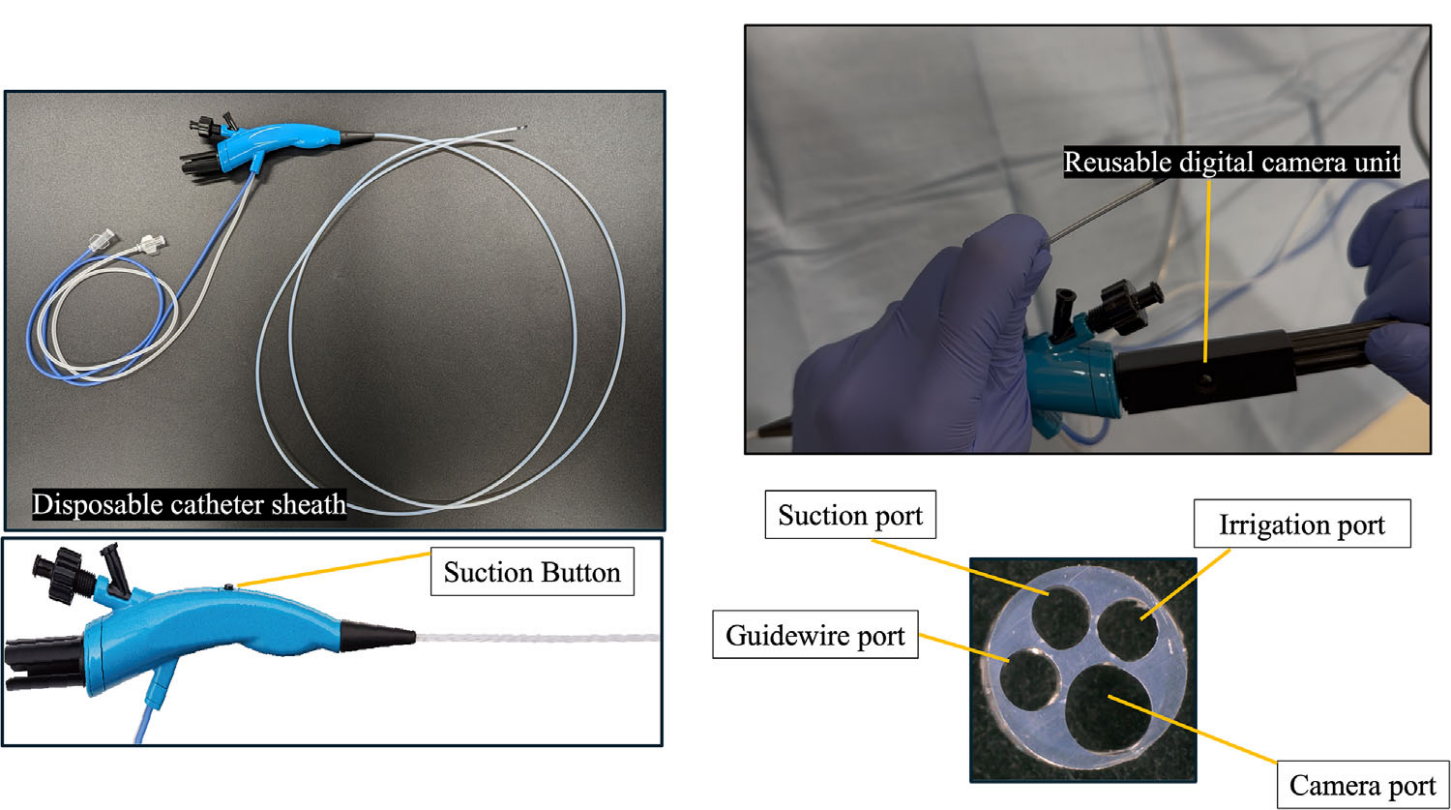
Ultraslim catheter‐type peroral cholangioscope/pancreatoscope (UCPOCS/UCPOPS). The external appearance and structural components of the UCPOCS/UCPOPS system (DRES Slim Scope; Japan Lifeline, Tokyo, Japan) are shown. The major components, including the reusable digital camera unit, disposable catheter sheath, guidewire channel, irrigation port, and suction lumen, are labeled for clarity. The guidewire channel enables over‐the‐wire insertion, whereas irrigation and suction functions facilitate maintenance of adequate visualization during intraductal observation.

The UCPOCS/UCPOPS system is an ultraslim catheter‐type cholangioscope/pancreatoscope designed to facilitate direct intraductal visualization of narrow bile and pancreatic ducts. Compared with conventional cholangioscopes and pancreatoscopes, this device has a markedly reduced caliber, allowing for easier insertion and improved accessibility in small‐caliber ducts and anatomically challenging situations. The insertion tube has a maximum outer diameter of 2.6 mm, with a distal‐end outer diameter of 2.3 mm. The effective working length is 1950 mm, enabling its use during standard ERCP and balloon enteroscopy‐assisted procedures in patients with surgically altered anatomy. This device is compatible with guidewires with a diameter of up to 0.025 inches and incorporates a guidewire channel with a minimum diameter of 0.68 mm, enabling over‐the‐wire insertion and stable advancement into the target duct (Table 1). Despite its ultraslim profile, the UCPOCS/UCPOPS system is equipped with irrigation and suction functions, allowing for sufficient field clearing during intraductal observation. However, the catheter‐type design does not permit active tip deflection, and maneuverability relies primarily on guidewire guidance and scope advancement/withdrawal. Additionally, although the guidewire lumen theoretically permits the passage of slim accessories, the channel diameter does not allow reliable biopsy or therapeutic device insertion; therefore, the present use of UCPOCS/UCPOPS is limited to diagnostic observation and procedural assistance rather than direct therapeutic intervention (Figure 1). Regarding image quality, the UCPOCS/UCPOPS system provides direct digital intraductal visualization with sufficient resolution for ductal inspection in narrow anatomical spaces. However, dedicated image‐enhancement technologies are currently limited S1.

**TABLE 1:** Technical specifications and features of the ultraslim catheter‐type peroral cholangioscope/pancreatoscope (UCPOCS/UCPOPS).

	Maximum diameter of the insertion tube	Distal End Outer Diameter	Compatible guide wire	Minimum diameter of the guidewire channel	Effective length
Device	2.6 mm	2.3 mm	≦ 0.025 inch	0.68 mm	1950 mm

### 2.2 Study Endpoints and Procedural Details

The primary endpoint was technical success, defined as successful scope insertion into the target duct. The secondary endpoint was clinical success. In diagnostic cases, clinical success was defined as the achievement of a definitive diagnosis or successful localization of the target lesion based on direct visualization. In procedural utility cases (e.g., ENGBD guidance), clinical success was defined as the successful achievement of the intended procedural goal. Diagnostic and procedural utility cases were analyzed separately. Adverse events (AEs) were assessed and graded according to the American Society for Gastrointestinal Endoscopy lexicon and Cotton severity classification [7].

Antibiotic prophylaxis was administered in four patients at the attending endoscopist's discretion, whereas therapeutic antibiotics were administered in two patients with inflammatory conditions. Ductal access in patients with surgically altered anatomy was achieved via short‐type balloon‐assisted enteroscopy‐assisted ERCP using a short‐type balloon enteroscope platform.

The two cases of post‐hepatectomy biliary bleeding and endoscopic naso‐gallbladder drainage (ENGBD) for acute cholecystitis included in the present series were previously described in case reports from our institution. These two cases were included in the current analysis to comprehensively evaluate the overall feasibility and safety profile of UCPOCS/UCPOPS in real‐world clinical settings.

Duct diameter was retrospectively estimated from fluoroscopic ERCP images.

## 3 Results

Twelve patients (nine men and three women) with a mean age of 68 years (range: 55–83 years) were included in this study. The UCPOCS was used in eight patients for the following indications: common bile duct or intrahepatic stones (*n* = 3), biliary strictures (*n* = 2), intrahepatic cholangiocarcinoma (*n* = 1), recurrent bile duct bleeding after hepatectomy (*n* = 1), and acute cholecystitis requiring ENGBD (*n* = 1). The UCPOPS was used in four patients for postoperative pancreatic duct evaluation (*n* = 2, including one patient after pancreatoduodenectomy) and IPMN evaluation (*n* = 2). Two of the included cases have been previously reported as individual case reports. These cases were re‐analyzed as part of the consecutive case series to evaluate the overall performance of the device. (Supplementary Table S1).

Successful scope insertion was achieved in patients with surgically altered anatomy via balloon‐assisted ERCP using a short‐type enteroscope. Antibiotics were administered to six patients (50%). No significant difference in the incidence of AEs was observed between patients who received and did not receive antibiotics. In this study, three cases underwent UCPOCS/UCPOPS without prior endoscopic sphincterotomy (non‐EST). These included one case with a native papilla, one case post‐pancreaticoduodenectomy (PD), and one case post‐endoscopic papillectomy (post‐EP).

The technical success rate was 100% (12/12). Overall clinical success was achieved in 11/12 cases (91.7%). Diagnostic clinical success was achieved in 10/11 diagnostic cases (90.9%), whereas procedural utility success was achieved in 1/1 procedural utility case (100%). One patient (8.3%) in the UCPOPS group developed mild post‐ERCP pancreatitis, which resolved with conservative management. No AEs, including cholangitis, were detected (Table 2).

**TABLE 2:** Patient characteristics and procedural indications.

	*n* = 12
Age (mean ± SD), years	68.1 ± 13.1
Sex (M/F)	9/3
UCPOCS/UCPOPS	8/4
diagnostic objective/therapeutic objective	11/1
Anatomy (normal anatomy/surgically altered anatomy)	10/2
Scope (duodenoscope/short SBE)	11/1
Antibiotics, *n* (%)	6 (50)
Antithrombotic agent use, *n* (%)	2 (16.7)
**Indications**	
UCPOCS (*n*)	*n* = 8
Stones	3
Strictures	2
Tumor evaluation	1 (Post‐EP)
Bleeding	1
Cholecystitis	1
UCPOPS (*n*)	*n* = 4
Stones	2 (Post‐PD; 1)
IPMN	2

Abbreviations: EP, Endoscopic papillectomy. IPMN, intraductal papillary mucinous neoplasm. PD, pancreaticoduodenectomy. SBE, single‐balloon enteroscopy.

In the post‐hepatectomy biliary bleeding case, the UCPOCS enabled the direct identification of the bleeding bile duct branch for the first time. An endoscopic nasobiliary drainage (ENBD) tube was placed at the bleeding site. On the same day, transarterial embolization was successfully performed with the ENBD tube as a radiological landmark, resulting in effective hemostasis [8].

In the acute cholecystitis case, the UCPOCS was used to assist in ERCP‐guided ENGBD. Direct visualization of the cystic duct orifice within the common bile duct facilitated accurate guidewire advancement into the cystic duct, enabling successful gallbladder drainage [9].

**FIGURE 2:**
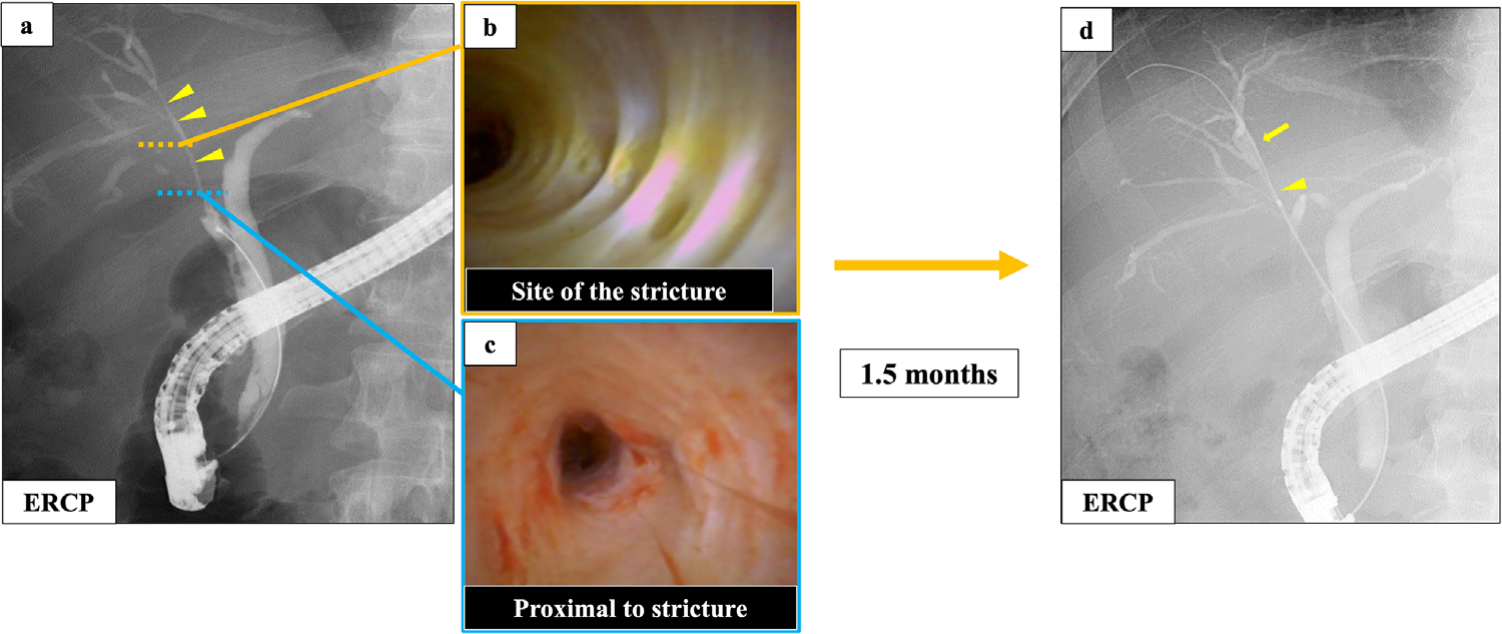
Ultraslim catheter‐type peroral cholangioscope (UCPOCS) findings in a patient with progressive biliary stricture. The UCPOCS was used in a male patient in his 60s to evaluate an indeterminate biliary stricture. (a) Endoscopic retrograde cholangiopancreatography (ERCP) revealed a long stricture in the right anterior segmental bile duct (yellow arrowheads), and the UCPOCS was advanced into the target duct. (b) The UCPOCS image at the stricture site revealed a wavy bile duct configuration; however, the mucosal surface appeared smooth and intact without apparent malignant features. (c) The UCPOCS image obtained proximal to the stricture showed mild erythema, possibly related to the guidewire or device contact, without gross mucosal abnormalities. Targeted biopsies were performed from the stricture and adjacent areas, and histopathological examination revealed inflammatory changes without any evidence of malignancy. (d) Follow‐up ERCP at 1.5 months later revealed the progression of the biliary stricture (yellow arrowheads) with upstream bile duct dilatation (yellow arrows). Subsequent biopsy specimens obtained from the stricture revealed an adenocarcinoma. This case highlights a potential limitation of visual diagnosis alone and underscores the importance of histological confirmation and follow‐up evaluation in lesions suspicious for malignancy.

Importantly, the UCPOCS may fail to facilitate the establishment of a definitive diagnosis in some cases, particularly in early‐stage or flat neoplastic lesions. This case highlights a clinically important limitation of visual assessment alone, as apparently benign mucosal findings did not exclude underlying malignancy. In our series, one patient initially showed only inflammatory changes on UCPOCS; however, follow‐up evaluation at 1.5 months later revealed bile duct adenocarcinoma requiring right hepatectomy (Figure 2), underscoring the need for short‐term follow‐up and, when appropriate, repeat endoscopic evaluation to minimize false‐negative diagnoses in high‐risk patients.

The median total procedure time was 42 (range: 25–69) min, and the median scope insertion time was 10 (range: 2–31) min. The median ductal diameter at the insertion site was 2.59 (range: 1,47–3.83) mm, indicating that the ultraslim device could be advanced even through ducts narrower than its own outer diameter (Table 3).

**TABLE 3:** Procedural outcomes and adverse events.

	*n* = 12
Technical success rate (%)	100 (12/12)
Overall clinical success (%)	91.7 (11/12)
Diagnostic clinical success (%)	90.9 (10/11)
Procedural utility success (%)	100 (1/1)
AE rate (%)	8.3 (1/12)
Pancreatitis (mild)	1
Total procedure time (median [range]), min	42 [25–69]
UCPOCS/UCPOPS insertion time (median [range]), min	10 [2–31]
Minimum duct diameter (median [range]), mm	2.59 [1.47–3.83]

Abbreviation: AE, adverse event.

Among the 12 patients, one patient with post‐hepatectomy biliary bleeding and one patient with acute cholecystitis requiring ENGBD were previously described in single‐case reports from our institution. All data were re‐evaluated and included in the present case series analysis.

## 4 Discussion

The present case series showed that UCPOCS/UCPOPS may provide clinically meaningful utility in diagnostic pancreatobiliary endoscopy, particularly in patients with narrow ducts or surgically altered anatomy. Conventional peroral cholangioscopes typically have outer diameters of approximately 4 mm, limiting stable insertion and visualization in patients with small intrahepatic branches or complex postoperative anatomy. In contrast, the 2.3‐mm UCPOCS/UCPOPS enables intraductal access to ducts frequently impassable with standard devices, thereby expanding the applicability of direct intraductal visualization.

Beyond feasibility, improved ductal access may enhance diagnosis in clinical scenarios in which fluoroscopy alone is insufficient, such as subtle mucosal changes, short strictures, or early‐stage lesions. Tonozuka et al. demonstrated the utility of a novel ultrathin peroral cholangioscope and reported that intraductal findings directly supported the diagnosis of benign biliary strictures [10]. These findings support the role of ultraslim cholangioscopy in improving diagnostic stratification of indeterminate biliary disease.

A particularly important advantage of direct intraductal visualization is its ability to localize the bleeding source in hemobilia or postoperative biliary bleeding. Shionoya et al. described a case of post‐hepatectomy biliary bleeding at our institution, in which a novel ultrathin peroral cholangioscope enabled identification of the responsible biliary branch when conventional ERCP findings were nondiagnostic [8]. The present case series included this previously described patient. In this case, UCPOCS enabled precise identification of the bleeding branch duct, and the ENBD tube placed at the bleeding point served as a radiological landmark for same‐day transarterial embolization, facilitating accurate hemostasis. This case illustrates that ultraslim intraductal endoscopy may contribute not only diagnostically but also to multidisciplinary coordination and procedural precision in complex biliary bleeding scenarios.

The procedural usefulness of UCPOCS beyond lesion inspection has also been demonstrated. In a patient with acute cholecystitis requiring ENGBD, direct visualization of the cystic duct orifice enabled accurate guidewire advancement into the cystic duct, improving the feasibility of transpapillary gallbladder drainage. This patient was previously reported by Hirakawa et al. from our institution, in which a UCPOCS facilitated cystic duct identification and successful ENGBD. The present series included this case, further supporting the practical value of ultraslim intraductal visualization as an adjunct during technically challenging gallbladder drainage procedures.

Another important advantage of slim intraductal endoscopy platforms is their applicability in patients with surgically altered anatomy. In this setting, intraductal visualization has become increasingly feasible despite complex postoperative anatomy. Tanisaka et al. reported the usefulness of a novel slim pancreatoscope during balloon enteroscopy for IPMN inspection via the papilla in patients with surgically altered anatomy [6]. Furthermore, Tanisaka et al. demonstrated the usefulness of a slim cholangioscope as an adjunct for biliary cannulation after Roux‐en‐Y gastrectomy [11]. Together with our experience, these findings suggest that reduced‐caliber intraductal endoscopy broadens diagnostic and procedural options for postoperative pancreatobiliary anatomy.

With respect to safety, our AE profile was favorable, with only one case of mild post‐ERCP pancreatitis and no cholangitis. Previous systematic reviews and observational studies of POCS reported overall AE rates in the single‐digit range, with cholangitis and pancreatitis being the most common complications [2, 12, 13]. Although direct comparisons among cholangioscopy systems remain limited, our findings suggest that UCPOCS/UCPOPS can be incorporated into routine ERCP without an apparent increase in procedure‐related AEs.

The procedure time is an important consideration in daily clinical practice. In our series, the median total procedure time and scope insertion time were 42 and 7 min, respectively. For comparison, Imanishi et al. reported a median insertion time of approximately 21 min for digital single‐operator cholangioscopy (SpyGlass DS) in routine diagnostic settings [14], and a meta‐analysis associated cholangioscopy‐assisted lithotripsy with substantially longer procedure times [15]. Although indications and procedural complexity differ, these comparisons suggest potential efficiency advantages of the ultraslim catheter‐type system.

Antibiotic prophylaxis may be beneficial during cholangioscopy‐guided procedures because prolonged intraductal manipulation and irrigation can increase intrabiliary pressure and promote bacterial translocation. Minami et al. reported favorable outcomes of digital cholangioscopy‐guided procedures and identified a lack of prophylactic antibiotic administration as a risk factor for postprocedural fever, supporting the importance of appropriate periprocedural management [16]. In our study, postprocedural cholangitis rates did not differ significantly according to antibiotic use, suggesting that routine prophylaxis may not be necessary in all cases using the ultraslim catheter‐type cholangioscope. One possible explanation is the relatively short procedure and insertion times in our series, which may have limited intraductal pressure elevation. Additionally, the small outer diameter of UCPOCS/UCPOPS may permit more effective bile drainage around the scope, potentially reducing biliary stasis. Although antibiotic prophylaxis may be beneficial during cholangioscopy‐guided procedures, no definitive conclusion regarding its necessity or efficacy can be drawn from this small observational study. Therefore, our findings should be considered exploratory, and further studies are needed before making procedural recommendations.

Despite the benefits of direct visualization, false‐negative assessments remain possible with POCS. In our series, a patient initially considered to have inflammatory changes on UCPOCS was later diagnosed with intrahepatic cholangiocarcinoma, highlighting a limitation of visual diagnosis alone. Therefore, visual assessment should be interpreted alongside clinical suspicion, imaging findings, and histological confirmation whenever feasible, particularly in cases suspicious for malignancy.

This observation aligns with the findings of previous reports, underscoring the need for close surveillance and, when appropriate, repeat cholangioscopic evaluation to minimize false‐negative diagnoses in high‐risk patients when clinical suspicion persists [1, 2].

UCPOCS/UCPOPS should be regarded as a complementary rather than a replacement platform for conventional cholangioscopy systems such as SpyGlass DS. Conventional systems retain advantages, including biopsy/therapeutic accessory compatibility and image‐enhancement technologies. In contrast, UCPOCS/UCPOPS may facilitate access to anatomically restricted regions that remain difficult to evaluate even with slim cholangioscopes, including peripheral intrahepatic ducts, areas beyond severe strictures, the cystic duct, and nondilated pancreatic ducts. In these settings, ultraslim visualization may aid lesion inspection and selective guidewire placement. Future technological refinement may further expand its clinical applicability.

Although the guidewire lumen conceptually suggests the possibility of passing through certain treatment probes, such as laser fibers, reliable therapeutic or biopsy device insertion is not currently ensured. While cholangioscopy‐assisted treatment for large biliary stones or advanced interventions in postoperative anatomy using various cholangioscopy systems have been reported, further technological refinement is required to enable ultraslim platforms to support broader therapeutic applications. Therefore, future development of ultraslim POCS/POPS systems should prioritize improved accessory compatibility, particularly for tissue acquisition and therapeutic probes, while preserving the advantages of small‐caliber access.

Consistent with the evolving role of POCS described in recent reviews, including that by Tanisaka et al., the ultraslim design of UCPOCS/UCPOPS represents a further step toward the expansion of the applicability of intraductal endoscopy. Given that the current system primarily improves diagnostic access rather than therapeutic capability, continued refinement of accessory compatibility may allow ultraslim platforms to contribute more fully to both diagnosis and treatment in the future [17]. This study has several limitations. First, this was a small, single‐center retrospective pilot study conducted at an expert center, which may introduce selection bias and limit generalizability. Second, patient selection and procedural indications were based on clinical judgement, and no conventional cholangioscopy control group was available for direct comparison. Third, the small sample size precluded robust evaluation of rare AEs, antibiotic prophylaxis, and subgroup analyses. In addition, duct diameter was retrospectively estimated from fluoroscopic ERCP images; because measurements may vary with contrast injection pressure and imaging conditions, reported values should be interpreted as approximate estimates.

In conclusion, UCPOCS/UCPOPS may represent a feasible complementary platform for intraductal visualization in biliary and pancreatic diseases, particularly in narrow ducts and challenging anatomy. Although currently limited mainly to diagnostic observation and procedural assistance, it may provide utility in anatomically restricted situations that are difficult to access using conventional devices. Further technological refinement and larger prospective studies are warranted to better define its clinical role.

## Author Contributions

Hiroyuki Kojima and Takao Itoi contributed to the study's conceptualization and methodology. Hiroyuki Kojima, Atsushi Sofuni, Takayoshi Tsuchiya, Reina Tanaka, Ryosuke Tonozuka, Shuntaro Mukai, Kazumasa Nagai, Yukitoshi Matsunami., Hirohito Minami., Noriyuki Hirakawa, Kyoko Asano, Kento Shionoya, and Kazuki Hama were involved in investigation and data curation. Hiroyuki Kojima and Takao Itoi performed the formal analysis and validation. Hiroyuki Kojima prepared the figures and drafted the original manuscript. Shuntaro Mukai supervised the study and served as the corresponding author. All authors critically reviewed and edited the manuscript and approved the final version for submission.

## Funding

The authors have nothing to report.

## Ethics Statement

**Approval of the Research Protocol by an Institutional Reviewer Board**: The study protocol was reviewed and approved by the Institutional Review Board of Tokyo Medical University (approval number: T2024‐0098).

## Consent

The authors have nothing to report.

## Conflicts of Interest

Takao Itoi serves as a medical advisor for Japan Lifeline and is the Editor‐in‐Chief of DEN Open. Shuntaro Mukai is an Associate Editor of DEN Open. The other authors declare no conflicts of interest.

## Supporting information




**TABLE S1 Detailed case characteristics and procedural outcomes of UCPOCS/UCPOPS procedures**. This table summarizes patient demographics, disease indication, target duct or ductal context, anatomical background, procedural platform, estimated minimum duct diameter, and procedural details for each individual case.
